# Identification and Analysis of *MS5*^d^: A Gene That Affects Double-Strand Break (DSB) Repair during Meiosis I in *Brassica napus* Microsporocytes

**DOI:** 10.3389/fpls.2016.01966

**Published:** 2017-01-04

**Authors:** Xinhua Zeng, Xiaohong Yan, Rong Yuan, Keqi Li, Yuhua Wu, Fang Liu, Junling Luo, Jun Li, Gang Wu

**Affiliations:** Key Laboratory of Biology and Genetic Improvement of Oil Crops, Ministry of Agriculture, Oil Crops Research Institute of the Chinese Academy of Agricultural SciencesWuhan, China

**Keywords:** *Brassica napus*, male sterility, meiosis, microtubule, double-strand breaks

## Abstract

Here, we report the identification of the *Brassica*-specific gene *MS5*^d^, which is responsible for male sterility in *Brassica napus*. The *MS5*^d^ gene is highly expressed in the microsporocyte and encodes a protein that localizes to the nucleus. Light microscopy analyses have demonstrated that the *MS5*^d^ gene affects microsporocyte meiosis in the thermosensitive genic male sterility line TE5A. Sequence comparisons and genetic complementation revealed a C-to-T transition in *MS5*^d^, encoding a Leu-to-Phe (L281F) substitution and causing abnormal male meiosis in TE5A. These findings suggest arrested meiotic chromosome dynamics at pachytene. Furthermore, immunofluorescence analyses showed that double-strand break (DSB) formation and axial elements were normal but that DSB repair and spindle behavior were aberrant in TE5A meiocytes. Collectively, our results indicate that *MS5*^d^ likely encodes a protein required for chromosomal DSB repair at early stages of meiosis in *B. napus*.

## Introduction

Meiosis is a highly conserved developmental process that is essential for eukaryotic sexual reproduction and produces haploid cells for progeny formation. In flowering plants, microsporocytes undergo meiosis to produce microspores in the anther, and megasporocytes undergo meiosis to produce megaspores in the ovary. During anther development, microsporocytes initiate meiosis as they develop from primary sporogenous cells. During meiosis, two successive rounds of chromosomal segregation (meiosis I and meiosis II) occur after a single round of DNA replication, without an intervening S-phase. Meiosis I is a reductional division in which homologous chromosomes are separated from each other. Meiosis II is an equational division that results in segregation of sister chromatids (Ma, 2005). Both meiosis I and meiosis II are divided into prophase, metaphase, anaphase, and telophase.

Meiotic prophase I is a unique and highly organized process that includes sister chromatid cohesion (SCC), homologous chromosomal alignment, pairing, synapsis, and recombination (Hamant et al., 2006). Highly organized chromosomes and correct chromosomal architecture are critical to meiosis and sexual reproduction (Kleckner, 1996; Zickler and Kleckner, 1999). Previous studies in many organisms have suggested that the successful completion of homologous chromosome pairing is dependent upon the progression of meiotic recombination, which is universally initiated by the generation of double-strand breaks (DSBs; Franklin et al., 1999; Li et al., 2004; Pawlowski et al., 2004; Ronceret et al., 2009). In addition, strong and close connections between homologous chromosomal pairs is stabilized by a network of longitudinal and transverse protein fibers, which are termed the synaptonemal complex (SC; Moens, 1969; Westergaard and Wettstein, 1972; Gillies, 1975). The search for sequence homology prior to SC formation has been proposed to be facilitated by the presynaptic alignment of homologous chromosomes in many organisms (Zickler and Kleckner, 1999). A number of spontaneous or induced synapsis mutants have been reported in rice (Kitada and Omura, 1983; Kitada et al., 1983; Nonomura et al., 2004a,b; Yuan et al., 2009) and *Arabidopsis* (Caryl et al., 2000; Armstrong et al., 2002). Mutations in SC genes typically cause defects in the homologous pairing, synapsis, and univalent formation processes. Mutations in genes that are involved in meiotic chromosome function often show defects in SCC and chromosome pairing (Lam et al., 2005; Hamant et al., 2006), DSB formation and/or repair (Lohmiller et al., 2008), and chromosome condensation, finally resulting in abnormal homologous chromosome segregation (Cai et al., 2003; Houben et al., 2005; Andreuzza et al., 2015). For example, *Arabidopsis* plants transformed with an RNAi construct targeting *AtZYP1* exhibit delayed meiosis and an absence of pairing and synapsis in most meiocytes (Higgins et al., 2005). In *Arabidopsis*, loss of *SPO11-1* and/or *SPO11-2* (conserved type-II topoisomerase-like enzyme SPO11) caused defective meiotic DSBs, homologous pairing and synapsis (Grelon et al., 2001).

Rapeseed, a crop belonging to the *Brassica* genus that is cultivated worldwide, is an important vegetable oil source for human consumption. Male sterility is the most effective and economical pollination control system for utilization of heterosis in rapeseed breeding. Previous studies have described the genetic male gene *MS5*, which has three different alleles (*MS5*^a^, *MS5*^b^, and *MS5*^c^) that lead to observed differences in fertility in *Brassica napus* (Lu et al., 2013; Xin et al., 2016). Plants carrying the *BnMs5*^b^*BnMs5*^b^ or *BnMs5*^b^*BnMs5*^c^ genotypes were sterile, whereas plants homozygous for the *BnMs5*^c^ or *BnMs5*^a^ allele were fertile (Lu et al., 2013). Relative to the sequence of *MS5*^a^, an 8,115-bp Mutator-like transposable element (MULE) insertion was detected in *MS5*^b^. Loss of *MS5*^a^ function had no effect on normal meiotic chromosome configuration at leptotene, but it resulted in abnormal chromosome morphology and cytokinesis during the following stages in *MS5*^b^*MS5*^b^ microspore mother cells (MMCs; Xin et al., 2016). Functional analyses demonstrated that *MS5* was essential for homologous pairing in meiosis but not for the initiation of DNA DSBs. Further analysis revealed that different expression levels of *MS5* and its allelic variants caused differences in fertility (Xin et al., 2016).

The thermosensitive genic male sterility line TE5A is a spontaneous mutant from the *B. napus* inbred line TE5 (Zeng et al., 2014). The fertility of TE5A is normal at low temperature, but it shows complete male sterility and partial female sterility at temperatures above 20°C. Previous genetic analysis revealed that the male sterility of TE5A is controlled by a single dominant gene, *BntsMs* (equivalent to *MS5*^d^; Zeng et al., 2014). Using a map-based cloning approach, the *MS5*^d^ gene was successfully localized to a region containing 24 annotated genes (Zeng et al., 2014). In the current study, *MS5*^d^ was successfully identified based on the microsynteny between *Brassica* and *Arabidopsis* and was confirmed by *Agrobacterium tumefaciens*-mediated genetic transformation assays. Further analyses demonstrated that the *MS5*^d^ gene in the TE5A mutant caused defects in DSB repair. Thus, the identification of *MS5*^d^ will contribute significantly to understanding the molecular machinery of plant meiosis and hence to crop breeding.

## Materials and Methods

### Plant Materials and Growth Conditions

The *B. napus* lines TE5A and TE5 were obtained from the Oil Crop Research Institute of the Chinese Academy of Agricultural Sciences; we described TE5A in a previous study (Zeng et al., 2014). Mutant lines were planted in a greenhouse under 25°C day/15°C night temperature conditions. All transgenic plants were grown under similar growth conditions.

### Functional Verification of Candidate Genes

To verify the functions of candidate genes, a 3.9-kb candidate genomic fragment containing the entire *MS5*^d^ coding region along with its 1.2-kb upstream and 0.5-kb downstream regions was amplified using the primer pair ZT2-1-F/R (Supplementary Table S1) with Pfu DNA polymerase (Fermentas) and cloned into the pCAMBIA2300 binary vector (Hajdukiewicz et al., 1994). The construct was confirmed by complete sequencing, introduced into *Agrobacterium tumefaciens* GV3101 host cells and transformed into calli with the homozygous *MS5*^d^ genotype, as previously described (Dun et al., 2011). Transformed plants with roots were subsequently transplanted into experimental plots. Mature transgenic plants were assessed for male fertility/sterility. DNA from the transgenic plants was analyzed by PCR using primers targeting the coding sequences of 35S and NPTII present in the vector (primers NPT-F/R and 35S-F/R; Supplementary Table S1). Pollen grains from the anthers of transgenic plants collected before anthesis were stained with 1% iodine/potassium iodide solution (KI/I_2_). The stained pollen was observed under a microscope.

### Histological Analysis

Paraffin and plastic sections were prepared to analyze flower development. Buds from various flower developmental stages (1.0–3.0 mm) were fixed in a solution containing 50% ethanol, 5% glacial acetic acid, and 3.7% formaldehyde for 24 h at room temperature; the tissues were then washed twice in 70% ethanol and stored in 70% ethanol at 4°C. For TEM analysis, fresh anthers from wild-type and *MS5*^d^ mutant plants at various developmental stages were fixed in 2.5% (w/v) glutaraldehyde in 0.1 M phosphate buffer (pH 7.4). The analytical procedures were performed as previously described by Zhu et al. (2010) and Yi et al. (2010).

### RT-PCR and qRT-PCR Analysis of Gene Expression

Total RNA was isolated from various tissues and buds of TE5A and homozygous TE5 plant lines using a plant RNA extraction kit (TIANGEN^1^) as recommended by the manufacturer. RNA (5 μg) was synthesized to first-strand cDNA using the ReverTra Ace-a-First-Strand cDNA Synthesis Kit (TIANGEN^1^). The reverse-transcription products from various tissues were then used as templates for RT-PCR and qRT-PCR. qRT-PCR was performed in 96-well optical plates containing SYBR Premix (TIANGEN) on an OPTICON 2 PCR instrument (MJ Research). Actin was used as a control for normalization. The primers RT1-F/R were used for the detection of *MS5*^d^ transcripts (Supplementary Table S1). All reactions were performed in three independent experiments. To determine the full-length transcript of *MS5*^d^, 5’-RACE and 3’-RACE were performed using total RNA from young buds with the SMART RACE cDNA amplification kit (Takara; Supplementary Table S1).

### Preparation of Meiotic Chromosome Spreads

Chromosome spreads were prepared from inflorescences that had been fixed in Carnoy’s solution (ethanol:glacial acetic acid, 3:1, v/v). Anthers containing microsporocytes undergoing meiosis were incubated with 3% cytohelicase, 3% pectolyase, and 3% cellulase in citric acid buffer for 90 min at 37°C and were then washed three times in PBS. Squashes were made in 45% acetic acid. The microscope slides were frozen in liquid nitrogen, and the coverslips were then removed. The chromosome spreads on air-dried slides were stained with 4′,6-diamidino-2-phenylindole (DAPI) in an antifade solution (Vector), and images were captured using a DM2500 microscope equipped with a DFC420C digital camera system (Leica).

### Protein Localization

The full-length cDNA of *MS5*^d^ without its termination codon (TGA) was PCR-amplified using the PM-F and PM-R primers (Supplementary Table S1) and inserted into the pM999GFP vector obtained from Dr. Jian Xu (National Key Laboratory of Crop Genetic Improvement, Huazhong Agricultural University, Wuhan, China). A cyan fluorescent protein (CFP) fusion with the chaperone binding protein GHD7 was employed as a nuclear marker protein; this fusion protein was obtained from Qifa Zhang (National Key Laboratory of Crop Genetic Improvement, Huazhong Agricultural University). The fusion constructs were introduced into *Arabidopsis* protoplasts prepared from whole seedlings via PEG/calcium-mediated transformation (Yi et al., 2010). Fluorescence microscopy was performed using a confocal laser microscope.

### *In situ* Hybridization

Inflorescences undergoing meiosis were fixed in 50% FAA solution (50% ethanol, 5% glacial acetic acid, and 3.7% formaldehyde) for 16 h at 4°C before being washed twice with 70% ethanol. The tissue was dehydrated through a graded ethanol series, transferred into xylene, embedded in paraffin wax and sectioned at a thickness of 8 μm. The *MS5*^d^ cDNA fragment was amplified with the primers *in situ*-F/R (Supplementary Table S1) and was then ligated into the linearized TA cloning vector pEASY-T3 (TransGen Biotech). The antisense and sense probes were subsequently transcribed *in vitro* from the T7 and SP6 promoters using polymerase with digoxigenin RNA labeling reagents (Roche). RNA hybridization and immunological detection of the hybridized probes were performed according to the protocol of Deblock and Debrouwer (1993). Images were captured with a DM2500 microscope using a DFC420C digital camera system (Leica).

### Immunofluorescence

For immunofluorescence, *B. napus* anthers were harvested and fixed in 4% (w/v) paraformaldehyde for 30 min at room temperature. Anthers with microsporocytes at the appropriate meiotic stages were flattened onto poly-*L*-lysine coated slides. After cellular debris was removed, the slides were blocked in 1 × PBS containing 1% BSA for 60 min and then incubated overnight at 4°C in a moist chamber with different antibodies (monoclonal anti-α-tubulin antibody: Sigma MFCD00145891; anti-γH2AX polyclonal antibody: Trevigen 4418-APC-100; anti-BnASY: self-prepared antibody) diluted 1:200 in 1 × PBS containing 1% BSA. After three washes in 1 × PBS, the slides were incubated with DyLight 488-labeled goat anti-rabbit secondary antibody or DyLight 594-labeled goat anti-rabbit secondary antibody (1:1000 dilution) for 1 h. The chromosome spreads were then counterstained with DAPI in an antifade solution (Vector). For immunofluorescence analyses, primary images were captured using a confocal laser microscope.

## Results

### Mapping and Isolation of the *MS5*^d^ Gene

**FIGURE 1:**
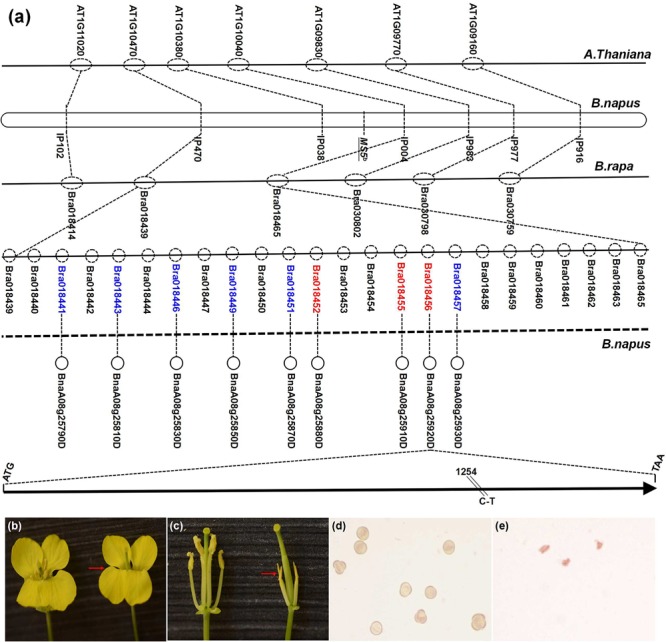
**Mapping and positional cloning of the *MS5*^d^ gene and phenotypic complementation testing with the *MS5*^d^ gene. (a)** The dotted circles indicate predicted genes of *Brassica rapa*; the red type indicates candidate genes that are not present in the corresponding *Arabidopsis* homologous region. **(b,c)** Flowers of a fertile non-transgenic TE5 plant (left) and of a sterile T_0_ transgenic plant in the TE5 background (right, red arrow). **(d)** Pollen grains of the non-transgenic TE5 plant stained with KI/I_2_ solution. **(e)** Pollen grains of the transgenic plant stained with KI/I_2_ solution.

The *MS5*^d^ gene had been previously localized to a 103-kb region that is highly syntenic to the *B. rapa* linkage group A8, and 24 putative genes (from *Bra018439* to *Bra018465*) were identified in the target region (**Figure 1a**) (Zeng et al., 2014). Based on homolog analyses in *Arabidopsis*, functional annotation of the *B. rapa* transcripts suggested that six of the 24 *B. rapa* genes (*Bra018457, Bra018451, Bra018449, Bra018446, Bra018443*, and *Bra018441*) were involved in anther development^2^. In addition, sequence comparisons revealed that three other *B. rapa* genes (*Bra018452, Bra018455*, and *Bra018456*) lacked homologs in the *Arabidopsis* syntenic region (**Figure 1a**). Based on the microsynteny of homologs in the *B. napus* linkage group N8 target region, these nine *B. rapa* genes were selected as candidate genes for *MS5*^d^. Next, we performed comparative sequence analysis of the nine candidate genes in the normal fertile line TE5 and the male sterile line TE5A. Interestingly, *BnaA08g25920D* (homologous to *Bra018456*) was the only gene in which a single-nucleotide polymorphism (SNP) was detected between TE5 and TE5A; no sequence differences were found in the other candidate genes (**Figure 1a**). Thus, we selected *BnaA08g25920D* as the target candidate gene.

To validate the candidate gene, functional verification experiments were performed by transforming wild-type *B. napus* lines with the full genomic fragment containing *BnaA08g25920D* from TE5A; the fragment contained a 1.2-kb native promoter region, the-1.39 kb *MS5*^d^ coding region, and a 1.41-kb downstream region. The amplified sequence was cloned into the pCAMBIA2300 binary vector, and the complementation construct was introduced into TE5 and Zhongshuang11 (a *de novo*-sequenced rapeseed cultivar in China) via *Agrobacterium tumefaciens*-mediated transformation. Of 34 kanamycin-resistant T_0_ transgenic TE5 plants, 23 lines exhibited the wild-type fertile flower phenotype, and 11 lines showed a male sterile phenotype at temperatures above 20° (**Figures 1b–e**). In addition, in the Zhongshuang11 line, three out of nine kanamycin-resistant T_0_ transgenic plants showed a male sterile phenotype. The recapitulated male sterility of the transgenic TE5 and Zhongshuang11 T_1_ progeny plants was co-transmitted stably, and it co-segregated with the introduced DNA. These experiments indicated that the male sterile phenotype of TE5A was caused by the SNP in *BnaA08g25920D*, which is equivalent to *MS5*^d^.

### *MS5*^d^ Is Highly Expressed in Microsporocytes and the Tapetum during Anther Development

**FIGURE 2:**
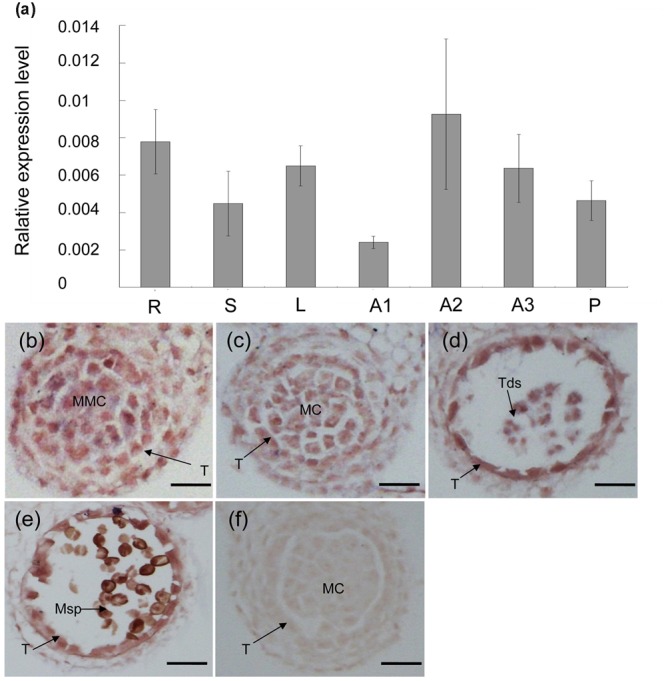
**Expression analysis of the *MS5*^d^ gene. (a)** Real-time PCR analysis of *MS5*^d^ in roots (R), leaves (L), stems (S), and young anthers (A) of TE5A. Young anthers of TE5A showed complete male sterility when grown at high temperatures (>20°C) (A1) and complete male fertility when grown at low temperatures (<20°C) (A2). Young anthers of the wild-type (A3) and pods (P) were also analyzed. Actin was used as a control for normalization. The data shown represent the mean ± SE of three independent experiments. **(b–f)** *In situ* hybridization assays of *MS5*^d^ in a longitudinal section at the young anther stage and in transverse sections at the early meiosis stage **(b)**, the meiosis stage **(c)**, the tetrad stage **(d)**, and the pollen mitosis stage **(e)**. **(f)** Negative control with sense probe in anthers at the meiosis stage. T, tapetum; MMC, microspore mother cell; MC, meiotic cell; Tds, tetrads; Msp, microspore; bars = 25 μm.

Various organs of TE5A plants were examined using real-time PCR to reveal the expression profile of the *MS5*^d^ gene. **Figure 2a** shows that *MS5*^d^ is expressed in various organs, including the roots, stems, leaf, pods, and young anthers. Expression of *MS5*^d^ was detected in the anthers of TE5 and TE5A plants at both the permissive and restrictive temperatures. To further investigate the spatial and temporal patterns of *MS5*^d^ expression during anther development, RNA *in situ* hybridization was performed using an *MS5*^d^-derived probe with sections of wild-type flower buds at different stages. In stages 5–12, *MS5*^d^ RNA was clearly detectable in the tapetum, microsporocytes, tetrads, and microspores. Maximal expression was observed in the tapetum and microspores at stage 8 (**Figures 2b–e**). A sense probe was used as the negative control (**Figure 2f**). These results suggest that *MS5*^d^ is highly expressed in microsporocytes during meiosis in the stamen of *B. napus*.

### *MS5*^d^ Encodes a Novel Protein Located in the Nucleus

**FIGURE 3:**
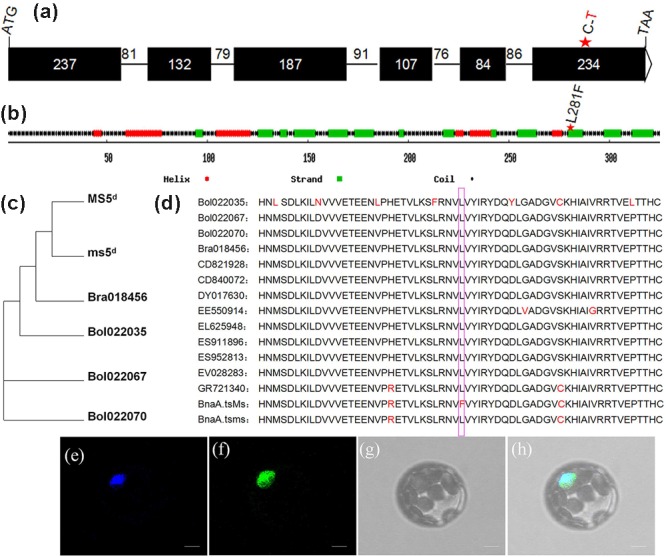
**Sequence analysis and subcellular localization of the MS5^d^ protein. (a)** Exon/intron structure of *MS5*^d^, with six exons (black boxes) and five introns; their corresponding sizes are shown. **(b)** Deduced MS5^d^ protein structure. **(c)** Phylogenetic analysis between *MS5*^d^ homologs identified in *Brassica oleracea* and *B. rapa*. **(d)** Deduced amino acid sequences of *MS5*^d^ homologs from different *Brassica* species were aligned with MEGA 4.0. The pink box indicates the L281F mutation. **(e)** The cyan fluorescent protein (CFP) signal was distributed throughout the protoplast cell after transformation with the control CFP construct. **(f)** The protoplast showed a green fluorescent signal after transformation with the MS5^d^-GFP fusion construct. **(g)** The same protoplast as **(e)** under bright field. **(h)** The images in **(e–g)** were merged. Bars = 10 μm in **(e–h)**.

By performing alignments to the *B. napus* genome database (Chalhoub et al., 2014^3^) and the *Brassica* database (BRAD^4^), the full-length coding sequence of *MS5*^d^ was predicted to comprise 981 bp and to consist of six exons (**Figure 3a**). This predicted exon-intron structure was experimentally supported by 5’-RACE and 3’-RACE analyses and RT-PCR product sequencing. *MS5*^d^ encodes a protein containing 326 amino acids with an estimated size of 37.8 kDa. The structure of the MS5^d^ protein was predicted using network protein sequence analysis^5^, which indicated that it contains six helices, 19 coils, and 13 strands (**Figure 3b**). The SNP (C-to-T) in the *MS5*^d^ gene occurred in the sixth exon and caused a substitution of Leu with Phe at position 281 (hereafter referred to as L281F) of the protein (**Figure 3b**). The substitution was located near the C-terminus of MS5^d^, and we speculated that the L281F mutation might modify the function of MS5^d^.

To investigate the subcellular localization of MS5^d^, we constructed a fusion protein of MS5^d^ with green fluorescent protein (GFP) driven by the 35S promoter. As a positive control, the previously characterized protein GHD7, which is located in the nucleus, was fused to CFP (Xue et al., 2008). The GHD7-CFP and MS5^d^-GFP fusion constructs were simultaneously introduced into *Arabidopsis* protoplasts via PEG/calcium-mediated transformation. The GFP signal colocalized with the CFP signal, indicating that MS5^d^ is a nuclear protein (**Figures 3e–h**).

To understand the origin of *MS5*^d^, BLAST was used with the full-length cDNA sequence of *ms5*^d^ and public databases, including the *B. napus* genome database (Chalhoub et al., 2014), the *Brassica* database (BRAD^4^), the NCBI database^6^, and the Phytozome database^7^. The results showed that three copies of the gene are present in *B. napus* (*BnaA08g25920D, BnaC08g14090D*, and *BnaC08g14440D*). In comparison, one copy was found in *B. rapa* (*Bra018456*), and two copies were found in *B. oleracea* (*Bol022067* and *Bol022070*). Interestingly, with the exception of homologs from *B. rapa, B. oleracea*, and *B. napus, ms5*^d^ yielded no homologs in other *Brassicaceae* species. Moreover, *ms5*^d^ displayed higher sequence identity with *Bra018456* (96.5%) than with the other homologs (**Figure 3c**). Despite the observed sequence divergence, the MS5^d^ L281F substitution was highly conserved in all of the analyzed *Brassicaceae* species (**Figure 3d**). Furthermore, a BLASTp search^6^ of the MS5^d^ protein sequence against public databases also uncovered no proteins with functional similarity in other organisms, except in the *Brassica* genus. Thus, we propose that the *ms5*^d^ gene, encoding a novel nucleoprotein, originated from *B. rapa* after its divergence from *Arabidopsis* and the *Brassica* species.

### Defective Meiosis in TE5A Microsporocytes

**FIGURE 4:**
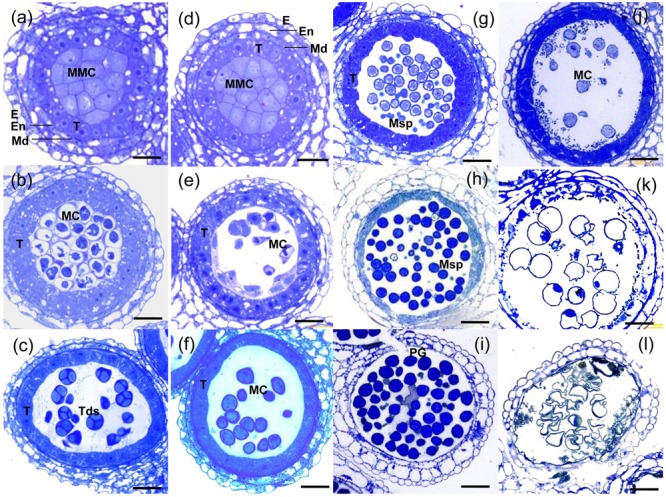
**Histological analysis of anthers from wild-type and TE5A plants. (a)** MMC stage of wild-type; **(d)** MMC stage of TE5A. **(b)** Meiosis of wild-type; **(e)** meiosis of TE5A. **(c)** Tetrad stage of wild-type; **(f)** tetrad stage of TE5A. **(g)** Vacuolated pollen stage of wild-type; **(j)** vacuolated pollen stage of TE5A. **(h)** Pollen mitosis stage of wild-type; **(k)** pollen mitosis stage of TE5A. **(i)** Mature pollen stage of wild-type; **(l)** mature pollen stage of TE5A. Arrows indicate the lipid bodies. E, epidermis; En, endothecium; ML, middle layer; T, tapetum; MMC, microspore mother cell; MC, meiotic cell; Tds, tetrads; Msp, microspore; PG pollen grain; Bars = 25 μm.

To investigate the anther development defects in the TE5A mutant when grown at high temperatures (>20°C), semi-thin sections of anthers were used to compare the differences between TE5A and wild-type TE5. No obvious differences were detected between the mutant and the wild-type during early pre-meiosis (stages 1–5). The anther primordia of both TE5A and TE5 differentiated to form the characteristic anther structure, with microsporocytes, locules, walls, connective tissues, and vascular regions characteristic of mature anthers (**Figures 4a,d**). At anther developmental stage 6, both the mutant and wild-type microsporocytes entered normally into meiosis (**Figures 4b,e**). At stage 7, the tapetal cell cytoplasm in TE5 became condensed, indicating that the tapetal cells were developing into secretory cells. The microsporocytes eventually completed meiosis to form tetrads that had characteristic callose walls (**Figure 4c**). In the TE5A anther, similarly to wild-type, the tapetal cells transformed normally into secretory cells at stage 7. However, the microsporocytes stalled in meiosis (**Figure 4f**). From stages 8 to 12 in the wild-type, after the callose wall was dissolved, the microspores were released into the locule. Subsequently, microspores underwent two rounds of mitosis and developed into viable pollen grains (**Figures 4g–i**). However, in TE5A, the microsporocytes did not undergo mitosis and then disintegrated concomitantly with the tapetum (**Figures 4j–l**).

**FIGURE 5:**
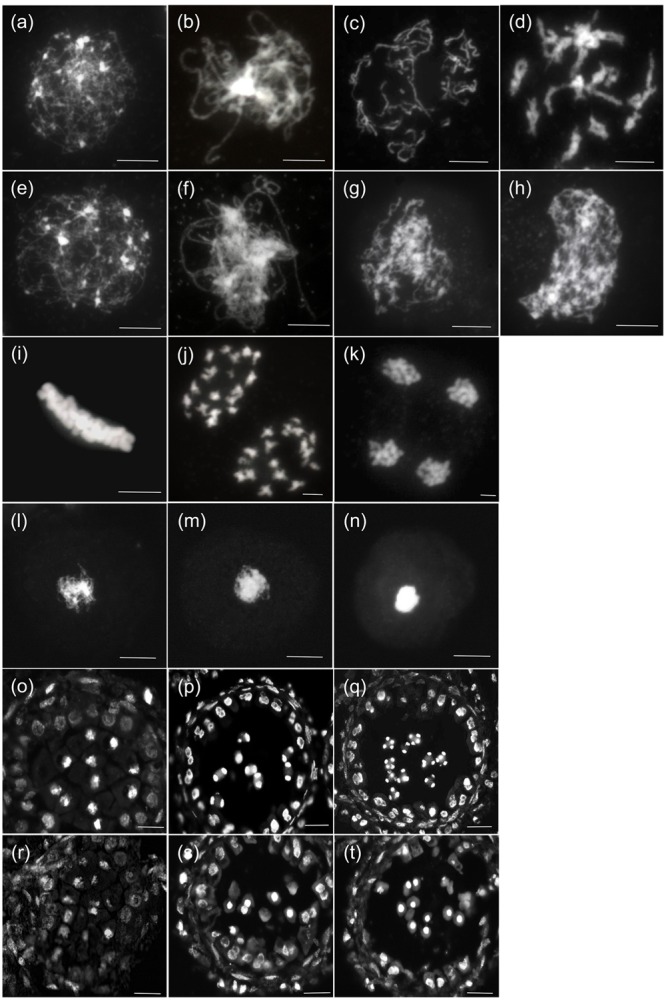
**Meiosis of wild-type and TE5A microsporocytes.** A male meiotic chromosome spread was stained with DAPI for the wild-type **(a–d),(i–k),(o–q)** and TE5A **(e–h),(l–n),(r–t)**. **(a,e)** Show leptotene; **(b,f,o,r)** show pachytene; **(c,g)** show diplotene; **(d,h)** show diakinesis; **(i,l)** show metaphase I; **(j,m,p,s)** show telophase I; **(k,n,q,t)** show the tetrad stage. **(a–n)** Bars = 5 μm. **(o–t)** Bars = 20 μm.

Next, we investigated the detailed chromosome behavioral defects of TE5A microsporocytes at different meiotic stages using DAPI staining analysis. From leptotene to pachytene, no obvious differences were observed between chromosome spread preparations in microsporocytes from TE5A mutant and wild-type TE5 plants (*n* = 26; **Figures 5a,b,e,f,o,r**). At diplotene, the homologous chromosomes of TE5 microsporocytes remained associated through chiasmata following breakdown of synapsis (*n* = 12; **Figure 5c**). However, in TE5A, unassociated chromosomes were present, and no condensed chromosomes or chiasmata were observed (*n* = 18; **Figure 5g**). At diakinesis, the chromosomes in TE5 microsporocytes condensed to produce very short pairs, and 19 bivalents could be identified (*n* = 14; **Figure 5d**). Subsequently, the bivalents aligned on the equatorial plate at metaphase I (*n* = 21; **Figure 5i**). Next, the chiasmata were released, and the homologous chromosomes segregated at anaphase I (*n* = 17; **Figures 5j,p**). Tetrads were finally produced after completion of the second meiotic division (*n* = 12; **Figures 5k,q**). In contrast to the wild-type, these chromosome behaviors were not observed in TE5A. Instead, unassociated and incompact chromosomes were present at diplotene (*n* = 20; **Figure 5g**). Crescent-shaped chromatin was present at diakinesis (*n* = 30; **Figure 5h**), and the characteristic metaphase I stage was missing. Furthermore, unusual condensed chromatin was observed (*n* = 25; **Figure 5l**), and it subsequently became highly condensed to form chromatin bodies at the dyad (**Figures 5m,s**) and tetrad stages (*n* = 32; **Figures 5n,t**) before disintegrating at the microspore stage (*n* = 16; **Figure 4k**). Collectively, these results suggest that the defective meiosis in the TE5A mutant is due to obviously abnormal chromosome dynamics that affect subsequent homologous chromosome segregation.

### Meiotic Chromosomal Axial Elements (AEs) Are Not Affected in TE5A

**FIGURE 6:**
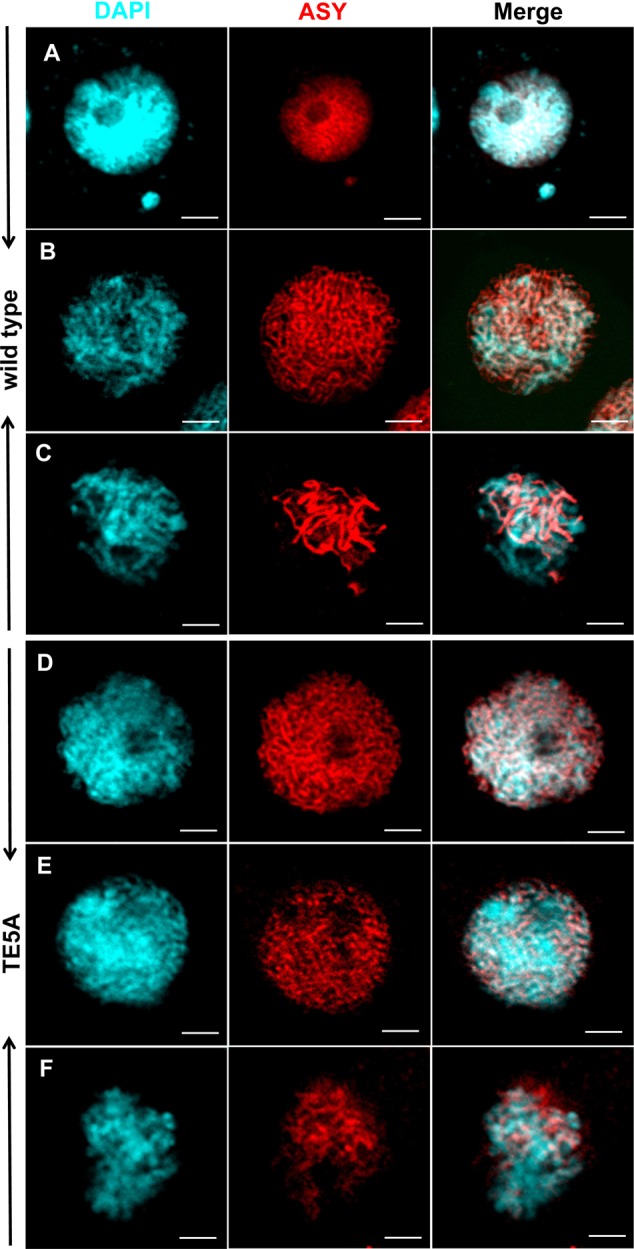
**Immunolocalization of ASY in wild-type and TE5A cells.** Immunolocalization using rabbit polyclonal antibody against ASY (red); chromosomal DNA was counterstained with DAPI (blue). Merged images show the overlap of blue and red fluorescence. **(A,D)** Show leptotene; **(B,E)** show early pachytene; **(C,F)** show pachytene. Bars = 5 μm.

The plant protein ASY1 is localized to the nucleus and associates with chromosomal axes to function as a good marker of early prophase I of meiosis (Caryl et al., 2000; Armstrong et al., 2002; Mikhailova et al., 2006). To investigate whether *MS5*^d^ affected AEs in TE5A, we performed immunolocalization analysis using a rabbit polyclonal antibody against the BnASY protein. The anti-BnASY antiserum clearly recognized both the recombinant protein and endogenous proteins extracted from the anthers of *B. napus* at the predicted 66-kDa size of BnASY (**Supplementary Figure S1**). Chromosomal axes in TE5 microsporocytes were first evident at leptotene (*n* = 25; **Figure 6A**), and short stretches of BnASY signals were present at early pachytene (*n* = 19; **Figure 6B**). Linear BnASY signals persisted until pachytene before disappearing as the homologs desynapsed (*n* = 20; **Figure 6C**). In TE5A, the immunolocalization signals appeared identical to those from leptotene to pachytene in TE5 microsporocytes (*n* > 15; **Figures 6D–E**), and the linear BnASY signals persisted until pachytene, when the chromatin started to disintegrate (*n* = 16; **Figure 6F**). The consistent distribution of BnASY1 along the chromosomal axes in both TE5A and TE5 microsporocytes indicates that meiotic AEs are not affected by *MS5*^d^ in microsporocytes during early meiosis.

### Meiotic DSBs and Microtubule Behavior in TE5A Microsporocytes during Meiosis I

**FIGURE 7:**
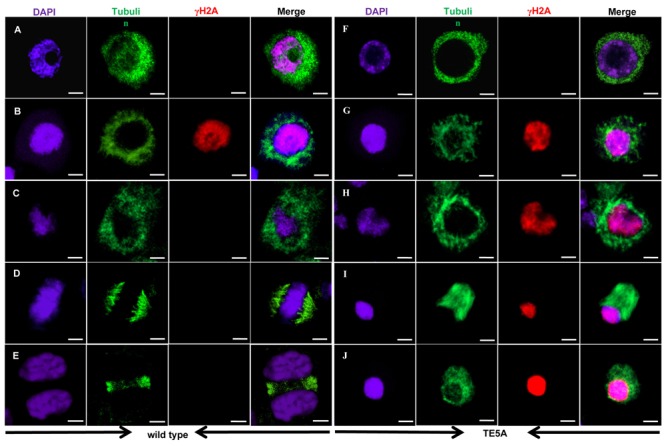
**Immunolocalization of tubulin and γ-H2AX in wild-type and TE5A cells.** Dual immunolocalization using a polyclonal antibody against tubulin (green) and rabbit anti-γ-H2AX antibody (Bethyl Laboratories) (red); chromosomal DNA was counterstained with DAPI (purple). Merged images show the overlap of green, purple, and red fluorescence. **(A,F)** Show leptotene; **(B,G)** show early pachytene; **(C,H)** show pachytene; **(D,I)** show metaphase I; **(E,J)** show telophase I. Bars = 10 μm.

4′,6-diamidino-2-phenylindole staining analysis suggested that the presence of the *MS5*^d^ allele resulted in the disintegration of chromatin after pachytene in TE5A microsporocytes. We thus sought to determine whether *MS5*^d^ affects microtubule dynamics and the process of meiotic DSB formation and repair. Previous studies have reported that deficiency in DSB formation is most easily detected by cytological detection of γH2AX rather than by the direct monitoring of DSB in DNA molecules (Sanchez-Moran et al., 2007). Thus, spindle morphogenesis and homologous DSBs were examined in meiosis I using dual immunostaining with anti-α-tubulin and anti-γH2AX antibodies (**Figure 7**). In TE5 microsporocytes, numerous diffuse γH2AX signals were detected throughout the chromatin at early pachytene (*n* = 13; **Figure 7B**), indicating DSB formation. In TE5A microsporocytes, γH2AX signals showed the same chronology and distribution at early pachytene as in TE5 (*n* = 18; **Figure 7G**). Thus, DSB formation appeared normal in TE5A microsporocytes. Following prophase I progression, the γH2AX foci disappeared at late pachytene in TE5 microsporocytes, suggesting that the DSBs were repaired normally (*n* = 17; **Figure 7C**). However, strong γH2AX signals were observed until the chromatin disintegrated in TE5A cells (*n* = 19; **Figures 7H–J**). Our observations indicate that DSB formation occurred normally in TE5A microsporocytes but that DSB repair progression failed at prophase I.

Furthermore, immunostaining analyses suggested that, in TE5 microsporocytes, a network of cytoplasmic microtubules was present around the nucleus at prophase I (**Figures 7A–C**). At metaphase I, the microtubules displayed an obvious bipolar and highly fusiform configuration, with the chromosomes located at the equator (*n* = 12; **Figure 7D**). At telophase I, the homologs of each bivalent segregated toward opposite poles, and the interzonal microtubules showed a tight configuration between the recently separated homologous chromosomes (*n* = 16; **Figure 7E**). In TE5A, the prophase network of cytoplasmic microtubules in microsporocytes was similar to that in TE5 microsporocytes from leptotene to pachytene (*n* = 20; **Figures 7F–H**). However, at metaphase I, the microtubules in TE5A microsporocytes showed a less clear-cut bipolar configuration compared to TE5, and they displayed a diffuse fusiform configuration (*n* = 29; **Figure 7I**). In addition, abnormal attachment between the chromosomes and microtubules was observed in TE5A microsporocytes (*n* = 20; **Figure 7I**). The microtubules then depolymerized and scattered throughout the nucleus (*n* = 30; **Figure 7J**). Collectively, these observations indicate that the attachment between the chromosomes and meiotic microtubules and the movement of microtubules are specifically defective in TE5A microsporocytes.

## Discussion

### The *MS5*^d^ Gene Functions in Gametophyte Development

This study identified a *Brassica*-specific gene, *MS5*^d^, and showed that it plays an important role in anther development. Our morphological and histological studies analyzing gametophyte development suggest that the *MS5*^d^ mutant shows complete male sterility owing to a failure in gametophyte development. Combined with evidence that the *MS5*^d^ gene is expressed in the anther during male meiosis (**Figure 2**), we conclude that *MS5*^d^ functions in male meiosis. Previous studies have reported that male sterility is often associated with defects in tapetum development (Jung et al., 2005; Li et al., 2006). Notably, the *PAIR3* gene, which is required for homologous chromosome pairing and synapsis in rice, is highly expressed in the degenerating tapetum. However, there was no obvious difference in the tapetum of the pair3 mutants, indicating that PAIR3 may not function in tapetal degeneration (Yuan et al., 2009). We observed a similar phenomenon in TE5A plants. Although the *MS5*^d^ gene was highly expressed in the tapetum, there was no obvious difference in tapetum development between TE5A cells (**Figure 4**) and the wild-type, indicating that *MS5*^d^ may not function in tapetal development. These results suggest that *MS5*^d^ has no function in parietal layer cell development.

### *MS5*^d^ Affects Chromosomal DSB Repair in *B. napus* Microsporocytes

The accurate segregation of homologous chromosomes at the first meiotic division depends on correct chromosomal behaviors, including SCC, homologous chromosomal alignment, pairing, synapsis, and recombination during prophase I of meiosis (Higgins et al., 2008). The formation and repair of homologous DSBs plays important roles both in maintaining genome stability and in generating genetic variability during the process of homologous recombination. The reciprocal exchange of DNA segments occurs between homologs after the repair of deliberately induced DSBs at prophase I and from random homolog segregation at anaphase I. In the present study, compared to the wild-type, TE5A meiotic chromosomes showed a normal configuration at leptotene and pachytene but displayed distinct chromosome morphology during the subsequent stages. Analyses of γH2AX signals suggested that meiosis-specific DSB formation was normal, whereas DSB repair was disrupted in TE5A microsporocytes, thereby causing the abortive segregation of homologous chromosomes. In *Arabidopsis*, several genes involved in the repair of *AtSPO11*-induced DSBs have been identified (Ma, 2006; Mercier and Grelon, 2008; Chang et al., 2011). Gene mutations, including *atrad50, atmre11, atrad51, atrad51c*, and *atxrcc3*, exhibited similar meiotic chromosome behaviors, such as asynaptic homologs and/or chromosome fragmentation (Bleuyard and White, 2004; Bleuyard et al., 2004; Li et al., 2004, 2005; Puizina et al., 2004). Unlike the defects observed in these mutants, the chromosomes in TE5A microsporocytes exhibited normal chromosomal AEs, as suggested by immunostaining for BnASY. Moreover, the γH2AX signals were relatively strong after late pachytene in TE5A, indicating that DNA damage may be more severe during the process of meiosis in TE5A. Taken together, these data raise the possibility that *MS5*^d^ controls DSB repair rather than DSB formation in *B. napus*.

### *MS5*^d^ Affects the Normal Attachment of Microtubules and Chromosomes in Male Meiosis I

Previous studies have shown that microtubules play a key role in telomere-mediated meiotic chromosome dynamics in a wide variety of organisms, including *Schizosaccharomyces pombe*, budding yeast (*Saccharomyces cerevisiae*), *Caenorhabditis elegans*, mice, rye, and wheat (Tepperberg et al., 1997; Ding et al., 1998; Yamamoto et al., 2001; Cowan and Cande, 2002; Cowan et al., 2002; Corredor and Naranjo, 2007; Sato et al., 2009). The separation of homologous chromosomes during anaphase I of meiosis is dependent on their correct association with the spindle. In the present study, microtubules showed abnormal movement and defective chromosomal attachment in TE5A microsporocytes. These results suggest that the *MS5*^d^ gene most likely affects the organization and movement of microtubules in *B. napus.* In *Arabidopsis*, the *mps1 atk1* mutant and *ATK1atk1*/*ATK5atk5* double heterozygote have been reported to be defective in chromosome segregation and spindle formation during meiosis (Chen et al., 2002; Quan et al., 2008; Jiang et al., 2009). In these mutants, homologous chromosomes are condensed and associate with the spindle normally but then separate asynchronously at the transition from metaphase to anaphase. However, in TE5A, the remarkable defects in chromosome dynamics included the lack of a characteristic diplotene, as suggested by the lack of chromosome condensation, a diffuse fusiform configuration of microtubules and disrupted attachment between chromosomes and microtubules. These findings indicate that the abortive homologous chromosome segregation in TE5A is significantly different from that in the *mps1 atk1* mutant or in the *ATK1atk1*/*ATK5atk5* double heterozygote. On the basis of these observations, we propose that mutation of *ms5*^d^ affects normal chromosome configuration, resulting in defective attachment between microtubules and chromosomes during *B. napus* meiosis.

### *MS5*^d^ and *B. napus MS5*^b^ Display Divergent Mechanisms Despite Sharing the Same Ancestor

Xin et al. (2016) demonstrated that the loss of the *MS5*^a^ gene resulted in abortive meiosis in the *B. napus* line Rs1046A; the phenotypic defects in microsporocytes resembled those of TE5A. We found that *ms5*^d^ and *MS5*^a^, both of which originated from Bra018456, exhibit the same sequences. Interestingly, an 8,115-bp MULE insertion into the *MS5*^a^ gene causes a recessive loss-of-function mutation known as *MS5*^b^, whereas an amino acid substitution in the C-terminus of *ms5*^d^ resulted in a gain of function in the male sterility gene *MS5*^d^. These findings suggest that *MS5*^d^ and *MS5*^b^ display divergent mechanisms but share a common ancestor. Functional divergence of genes from common ancestors can result in three distinct evolutionary fates: gene silencing with negative mutations, or pseudogenization; partition of ancestral function, or subfunctionalization; and acquisition of new function, or neofunctionalization (Force et al., 1999; Conant and Wolfe, 2008; Liu and Adams, 2010). Thus, we propose that *MS5*^d^ was derived from the neofunctionalization of its ancestor, whereas *MS5*^b^ originated from gene silencing with negative mutations of the same ancestor of *MS5*^d^ (Xin et al., 2016). These findings provide an interesting system in which to study the divergence of functional genes in paleoploid *Brassica* species.

A loss of function of the *MS5*^a^ gene caused by an 8,115-bp MULE insertion resulted in abnormal chromosome morphology and cytokinesis after the leptotene stage in MS5^b^MS5^b^ MMCs (Xin et al., 2016). *MS5*^d^, with an amino acid substitution in the C-terminus of *ms5*^d^, resulted in aberrant meiotic chromosome dynamics and pachytene arrest. We speculated that these differences might be caused by variations between *MS5*^b^ and *MS5*^d^. The loss of *MS5*^a^ gene function has been suggested to affect synapsis and homologous pairing in meiosis, but it has no detectable effect on ASY1 loading (Xin et al., 2016). In our study, a similar phenotype was observed by FISH (Yan et al., 2016) and anti-BnASY in TE5A. These results indicate that different mutations of *MS5*^b^ and *MS5*^d^ have no effect on chromosomal axis formation in meiosis. Further experiments are required to reveal the effect of *MS5*^d^ on synapsis of homologs in meiosis. Previous results suggested that *MS5* was essential for DSB repair, although it had little effect on DSB formation. In the present study, immunofluorescence experiments showed that *MS5*^d^ affected DSB repair but was dispensable for DSB formation. These results indicate that different mutations of *MS5*^b^ and *MS5*^d^ might have a similar effect on homologous recombination. Collectively, our results provide valuable information for understanding the molecular mechanism of *ms5*^d^ and the molecular machinery of plant meiosis.

## Author Contributions

XZ, XY, RY, and GW designed and supervised the study; YW, FL, and JLuo participated in its design. RY participated in the mapping and transgenetic experiments. XY, KL, and JLi participated in the cytology and histology analysis. XZ and GW wrote the manuscript. All the authors discussed the results and contributed to the manuscript. All authors read and approved the final manuscript.

## Conflict of Interest Statement

The authors declare that the research was conducted in the absence of any commercial or financial relationships that could be construed as a potential conflict of interest.

## Footnotes

1 http://www.tiangen.com/

2 http://www.arabidopsis.org/

3 http://www.genoscope.cns.fr/brassicanapus/data/

4 http://brassicadb.org/brad/

5 http://zhanglab.ccmb.med.umich.edu/

6 http://www.ncbi.nlm.nih.gov/

7 http://www.phytozome.net/

## Supplementary Material

FIGURE S1 **Western blot with BnaASY polyclonal antibodies**.
