# Supplementary figures and images for: Identification and Analysis of MS5d: A Gene That Affects Double-Strand Break (DSB) Repair during Meiosis I in Brassica napus Microsporocytes
Source: Front Plant Sci. 2017 Jan 4;7:1966. doi: 10.3389/fpls.2016.01966 (PMC5209369; doi:10.3389/fpls.2016.01966)

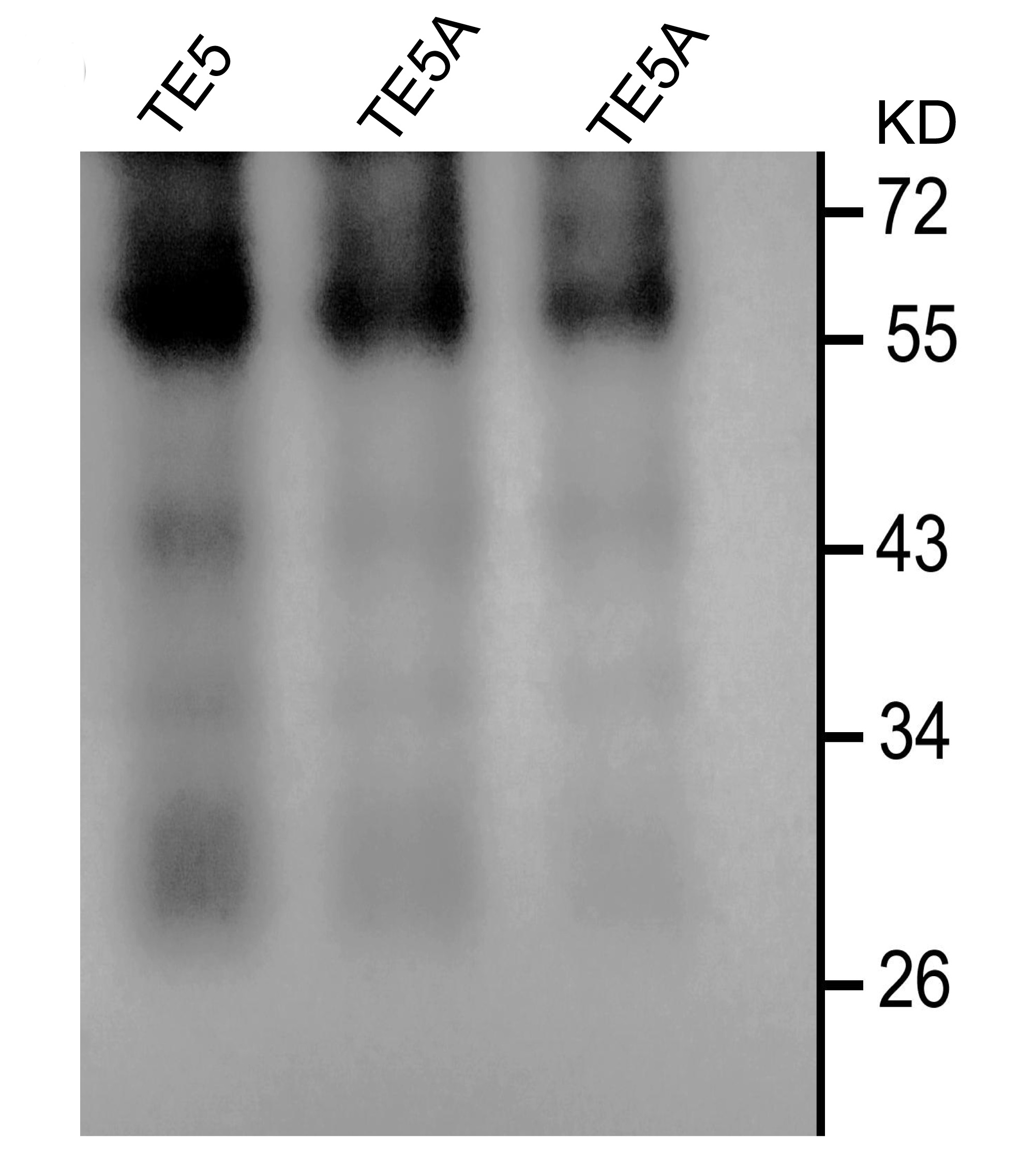

Supplement: FIGURE S1 — Western blot with BnaASY polyclonal antibodies. [file Image_1.JPEG]
