# Supplementary material for: Identification and Analysis of MS5d: A Gene That Affects Double-Strand Break (DSB) Repair during Meiosis I in Brassica napus Microsporocytes
Source: Front Plant Sci. 2017 Jan 4;7:1966. doi: 10.3389/fpls.2016.01966 (PMC5209369; doi:10.3389/fpls.2016.01966)
Supplement: Supplementary file 2 [file Table_1.DOC]

| Primer Name | Sequence |
| --- | --- |
| ZT2-1L | 5'-CCGGAATTCCTATTAATAAATTAATGACTCAGCT-3' |
| ZT2-1R | 5'-CAACTGCAGCCAAGAAGAGAATTGATTCCACA-3' |
| 5'RACE-1 | 5'-CCTCGGCCTTACGAATAGTCTCTTC-3' |
| 5'RACE-2 | 5'-CTTCTTTTCCAAGCGCCGATATG-3' |
| 3'RACE-1 | 5'-GTCGAGCCGACAACTCACTG-3' |
| 3'RACE-2 | 5'-GACGTAGTGGTAGAGACTGAGG-3' |
| RT-1L | 5'-AGCTACCTCCTCCTTTGTTGT-3' |
| RT-1L | 5'-CTCCTTTGAAGGCCACTTG-3' |
| PM-F | 5'-TGCTCTAGAATGAGTAATTCTGCGCCTGGAGGGT-3' |
| PM-R | 5'-TGCTCTAGACTCAGAGTCGGGCACAAGCTGAGAC-3' |
| insitu-F | 5'-TACGAATTCATGAGTAATTCTGCGCCTGGAGGGT-3' |
| insitu-R | 5'-CGGAAGCTTTCATACCACCAAAATAACAAGGAGC-3' |
| NPT-F | 5'-GGATCTCCTGTCATCT-3' |
| NPT-R | 5'-GATCATCCTGATCGAC-3' |
| 35S-F | 5'-AAGACATCCACCGAAGACTTA-3' |
| 35S-R | 5'-AGGACAGCTCTTTTCCACGTT-3' |

Table S1: The list of PCR primer that used in this paper.
